# The Hospital Nursing Supplement

**Published:** 1893-02-25

**Authors:** 


"The Hospital" Nursing Mirror.

Being the Extra Nursing Supplement of "The Hospital" Newspaper.

[Contributions for this Supplement should be addressed to the Editor, The Hospital, 140, Strand, London, W.C., and should have the word "Nursing" plainly written in left-hand top corner of the envelope.]

### News from the Nursing World.

### ROYALTY AT PORTSMOUTH.

The annual meeting of the Portsmouth Association for Nursing the Sick Poor was held on February 14th, and was honoured by the presence of their Royal Highnesses the Duke and Duchess of Connaught. The Duke moved a resolution commending the association, which is affiliated with the Q.V.J.I., to the sympathy of the people of Portsmouth. The resolution was seconded by Admiral the Earl of Clanwilliam. The Mayor presided at the meeting, which was well attended. For some years previous to the instituting of Queen's Nurses the work of attending on the sick poor of Portsmouth in their own homes, was managed by Miss Day and Miss Lawder, who have now taken up other work in which they will doubtless be equally successful.

### A PLEA FOR THE DOLLS.

The Nurse Dolls whose travels have been reported in The Hospital from time to time, have recently received an invitation to make their appearance in public again. This time they are asked to assist at a bazaar, and to lend their passive aid in increasing the funds needed for the establishment of a cottage hospital. The dolls do not appear unwilling to go, and doubtless even feel flattered at the prospect of serving a good object; but the Editor considers them quite unfit to pay a country visit at present. They inhabit cases fitted with glass doors, but London smuts and London dust have penetrated, and each month has added a deeper shade to our poor little nurses' garments. In fact, to use the plainest of English words, the dolls are dirty! They form a large family, as many of our readers know, and the groups of uniforms are of considerable interest, and it seems a great pity the little creatures should be permanently "out of work." Soap and water, needles and thread, ingenuity and a little patience, would soon restore their original attractions. Will our readers volunteer to help in this piece of work? If several parties of friends would each arrange to renovate a certain number of the dolls, we should soon be in a position to again lend out the collection to further worthy charitable objects. Suggestions and offers of help can be sent to Editor, The Lodge, Porchester Square.

### A PROMISING ASSOCIATION.

The Seaham Harbour Nursing Association, which was first started by the energy and generosity of Lord and Lady Londonderry, is already making its value felt. Nurse Hicks has been engaged for the current year, a month's probation having proved both need for and appreciation of her services. During those four weeks she visited 68 patients, and the council hope to supplement her ministrations by gifts of nourishing food and clothing, &c. Some of the members of the Executive Council are workmen, and the entire organisation appears to be of a most practical character.

### BRAVE NURSES.

The wooden building which served as an infectious hospital at Kidderminster was burned down the other day. One little child's life was sacrificed, but all the other patients were saved, mainly by the presence of mind of the Matron, who, with her nurses, did brave work on the occasion. At St. Luke's Hospital, New York, a catastrophe was recently averted by the common sense of two nurses. They found a large Christmas tree had caught fire, and they quietly closed all doors of communication, thus preventing the inmates of the wards from knowing of the accident, and by enveloping the blazing tree rapidly in blankets they controlled the flames until the hose was unrolled by the men of the establishment and brought swiftly to the spot.

### STAGNATION AT EASTVILLE.

The two lady guardians on the Barton Regis Board have so far failed in their praiseworthy efforts to obtain trained night nurses for the sick poor at Eastville Workhouse. Mr. Palmer and Mr. Cunningham moved and seconded the adoption of the report which "recommended that in face of the evidence given at the meeting of the committee, no such nurses should be appointed," says the *Bristol Mercury*. Miss Woollam, who explained so well at the previous meeting the absolute need for proper night attendants, begged that at least in the women's wards two nurses should be granted. "She believed the sick were in a worse condition than when she first visited the house, for then extreme cases were sent into the lunatic ward because there was a night nurse there." This is a revelation indeed of what the inmates of some infirmaries have to put up with. Our correspondent from East Dulwich tells us of extra help granted for bad cases in that beautiful infirmary, and we hope the same assertion can safely be made regarding other such institutions. Anything more terrible than for a person dangerously ill to be relegated to the companionship of lunatics merely to ensure the unfortunate patient getting some night attendance has seldom been reported. As the opposition to the proposed amendment of the two excellent lady guardians has now proved, the male members of the Barton Regis Board are determined that their sick and suffering brethren must in future be left entirely at the mercy of "a deputy attendant—one of the paupers," who "slept in the ward." Next time one of the gentlemen guardians is the victim of a serious illness it will be a matter of public interest to learn whether he is willing to have a sleeping pauper installed as his sole attendant during the long night watches. Charles Kingsley's "Madam Bedonebyasyoudid" would shed a light on this question.

### INTERESTING AND INSTRUCTIVE.

The Scotch Girls' Friendly Society at Boddam have recently had some capital class instruction on "Home Nursing, and the Treatment of Common Accidents and

Sudden Illness." Dr. Carmichael gave the lectures, and his tenth and last address was open to all girls and ladies in the district. The subject of it, "The Management of Infants and the Nursing of Sick Children," is assuredly one which cannot be sufficiently impressed on the mothers of the future. Many lives would be saved, and many delicate children would become strong ones, if domestic and personal hygiene formed part of every woman's education.

### KINDLY ENCOURAGEMENT.

One of the pleasantest sentences in the Bishop of Derry's speech at the meeting of the Londonderry District Nursing Society referred to the individual work of the nurses. His Lordship said that the public was able to do very little in return for what "the nurses do so well," and as he appealed for subscriptions to increase the work, he made plain to his hearers that donations of money were small matters in comparison with the personal service of true nurses. The workers at Londonderry are to be congratulated on the hearty appreciation shown to their labours.

### MODERN METHODS.

There is an interesting article in the *Morning Post*, Allahabad, on nursing progress in India. Speaking of the hospitals at Calcutta, Bombay, Madras, Lahore, Poona, &c., where large staffs of trained nurses are established at present, the writer draws a comparison between modern times and the days when "medical apprentices were required to do special duty in the wards of the big hospitals, such duty consisting in tending generally to the sick. In hospitals having no medical apprentices attached to them, patients were left to the tender care of native 'ward-boys.'" It is easy to see that the medical 'prentices reaped substantial advantages from experience thus gained, but probably the new order of things is much more agreeable to the sick people.

### AMERICAN NURSES.

In the *Trained Nurse* for February, Miss Helen Manning gives some capital hints on diet under the heading "Hygiene for Nurses." At the Rochester, U.S.A., Homœopathic Hospital Training School, the term of probation for the candidates is increased from one month to three. "The longer period," says the *Trained Nurses* "making it more certain that the applicant is qualified for the work she undertakes" in this, the Homœopathic Hospital has set an example which other institutions would do well to follow. At Rochester, U.S.A., the authorities of the Training School are anxious to have a proper Nurses' Home, and doubtless they will soon secure one, now that it is an acknowledged necessity. The Training School was founded twelve years ago, and the nursing staff has been increased from 12 to 33 members. In the course of a probationer's two years' training, she works in surgical, medical, maternity and children's wards, diet kitchen, night duty, private cases, out-patients, and district nursing, which sounds a long list indeed when we add to it theoretical instruction, lectures, and examinations.

### PROPOSALS IN PARIS.

The Syndicate of Infirmary Attendants and Nurses at Paris is anxious to bring about several reforms which appear greatly needed. The Municipal Council has received a petition on the following details, namely —The reduction of the working day to twelve hours (from six in the morning till six at night) with one hour for recreation; that vacancies in the hospital shall be made known at a central office, such as the Syndicate; that the dormitory system shall be abolished and separate bed-rooms provided for the nurses; that certificated attendants and nurses shall not be dismissed without serious cause, and then only after report to the Director of the Assistance Publique; also that certificated attendants who have already served in the hospitals shall be chosen for night duty in preference to inexperienced persons; the clothes and papers of the attendants shall be inspected only on their leaving the hospital; food shall be better prepared, and liberty given to bring in food from outside; scholarships reserved to the Schools of Attendants and Nurses shall be annually drawn by lot amongst those admitted. Finally, it is recommended that attendants and nurses shall be prohibited from receiving gratuities, and their salaries shall be increased.

### SHORT ITEMS.

The nurses at St. George's in the East Infirmary spent a very pleasant evening on the 8th inst. They first of all performed some tableaux vivants, which they had arranged with the kind assistance of the Matron; a musical entertainment and two clever recitations followed, and a little impromptu dance concluded a most successful "social evening."—The partially blind girl to whom Mrs. Creighton Hall gave free instruction in massage last year, is now earning a comfortable livelihood. Before taking up massage she was dependent on the small sums which she obtained by knitting, supplemented by a charitable grant. Now the young woman has achieved independence, and has been recently employed as a masseuse in U.C. Hospital.—Nurse Weedon has received Lewis's "Theory and Practice of Nursing" as her prize for Our Examination for February.—The Chertsey Board of Guardians have agreed to appoint a night nurse at the Infirmary.—At the Grahamstown Asylum systematic teaching of the nurses and attendants has been commenced by Mr. Greenlees, whose introductory lecture was an admirable one.—Miss Forsyth has received the badge of the Q.V.J.I. at Arbroath, where she has already won golden opinions by her excellent work.—The Morrison Prizes "For Meritorious Attendance on the Insane" have this year been awarded at Edinburgh to Thomas Cowan, of Sterling Asylum, and Isabella Brander, of Edinburgh.

### "THE HOSPITAL" ENDOWED BED.

Subscriptions for the Nurses' Bed are now due. All contributions sent to the Editor, The Lodge, Porchester Square, will appear in The Hospital. £1 acknowledged last week from A. B. should have been £1 1s. We have since received: 2s. from G. R. Jenkins, £1 1s. from Sister Lund, making total amount up to date £11 16s.

### A DISABLED NURSE.

It is not possible at present to contemplate a change of scene for the Disabled Nurse, as she is unable to bear any fatigue. We therefore propose to reserve £2, to be used for this purpose later on, making a weekly allowance of ten shillings to the invalid as long as the donations permit of it. We have this week received 2s. 6d. from Jane Gerrard, 10s. from Two Friends, and 4s. 6d. collected by Nurse Eleanor, making a total of £10 5s. 6d. Up to present date 27s. 6d. has been handed to Disabled Nurse, leaving balance of £8 18s.

### Management of Consumption.

### III.—HÆMOPTYSIS.

Hæmoptysis, or spitting of blood from the lungs or air passages, may be due to a variety of morbid processes going on in the respiratory organs or their appendages. It is always to be viewed with apprehension. Though almost every disease with which the lung may become affected is attended with hæmoptysis of trifling or considerable amount, yet by far the most common cause of this event is tubercular disease of the lung. A patient affected with tubercular phthisis scarcely ever goes through his disease without having hæmoptysis at some period of its course. So much is this the case that it has been estimated that between 80 and 90 per cent. of all the cases of phthisis have this complication. The amount brought up may be so small as only to streak the phlegm with which it is mixed, or so large that it may be measured by the pint or quart. Sometimes the result of such a profuse hæmoptysis may be death at once from choking, but this termination is rare; in the large majority of cases the patient recovers from his attack, and when he is blanched and has apparently lost far more blood than is compatible with life, the bleeding stops, and he slowly recovers strength again. The way in which an attack of hæmoptysis comes on varies; the patient may be lying in bed or walking about, or he may have been doing something requiring more physical exertion than he was fit for; he feels a tickling sensation in his throat, begins to cough, and is astonished and alarmed to find that each cough brings up a mouthful of bright red frothy blood. This may go on to such an extent that the blood pours from his nose and mouth; his anxiety and agitation become extreme, the pulse becomes quick, and a cold sweat breaks out on the forehead. Then, all being well, the bleeding stops; but for days afterwards the patient is in a weak condition, the expectoration continues stained with blood, and the temperature is raised to a higher level than it was before the attack. Sometimes the phthisis which had before been quiescent is lighted up as it were by the blood spitting and progresses at a great rate, in other cases no appreciable harm has been done.

Tubercular phthisis is a disease in which, by means of a specific organism—the tubercle bacillus—tubercles are set up in the lungs. These tubercles after invading the lung tissue break down, causing destruction of portions of the lung. The coats of the blood vessels in the lung become similarly involved and weakened, and under efforts, such as coughing, they rupture, or, owing to their weakened condition, a yielding takes place at some particular part of the vessel, and a minute aneurism or swelling is formed, which is very liable to give way. This explanation, a rottenness of pulmonary vessels produced by the effects of tubercle, accounts for the majority of cases of hæmoptysis. In some few cases congestion of a part of the lung is enough to bring it about. In a former paper stress was laid upon the importance of keeping a ward in which people with phthisis are living from becoming too hot, a limit somewhere between 58° and 62° being chosen as the proper temperature, and, with a view to the prevention of hæmoptysis, this rule of keeping the ward cool is particularly necessary. One frequently observes that in wards kept too hot hæmoptysis not only often occurs, but is extremely troublesome and difficult to treat, whereas in wards kept cool such an occurrence is rare, and when it does happen, presents little tendency to be lasting, and often easily succumbs to treatment. How much success will attend the treatment of a given case of hæmoptysis will very much depend upon the amount of judicious nursing the case has received. When a patient is seized with hæmoptysis he should, if not already in bed, at once be placed there, and while he is bringing up blood he may be allowed to sit up in bed; by so doing he can more readily get rid of the blood in his lungs, and there is less likelihood of its accumulating in such large quantities as to cause choking. As soon as the first sharp attack is passed he must lie down upon his back with the head and shoulders slightly raised by means of a soft pillow. The bed on which he rests should be firm and hard, and, if possible, made of horsehair. The covering should be light, and consist of no more than a sheet and a blanket. There should be no fire in the room, the windows should be slightly open, and the temperature of the room may vary from 55° to 58°. As the attack passes off the temperature can be raised to 60°, and another blanket may be allowed if the patient feels cold. When once the patient is in bed it is essential to his recovery to lie perfectly still; all unnecessary movement must be prohibited, and absolutely no talking allowed. In the weak state in which the hæmoptysis has left the patient, the slightest excitement, even going to the side of his bed, will increase the pulse rate and render him liable to another attack. So the nurse should perform her duties quietly and with as little inconvenience to the patient as possible. All attempts at thoroughly washing him must be abandoned, and on no account should his clothing be changed for the first few days. Many people are naturally upset and horrified at the sight of blood, and in nursing a case of hæmoptysis it is well to remember this, and to cover up any spots on the sheet or nightdress with a clean white cloth.

Next to attending to these small points the feeding of the patient should occupy the nurse's attention. The object in view is to give suitable food in such quantities as is necessary to keep the patient alive. He does not require much nourishment for the first few days, and the reason is this, he has swallowed a considerable amount of blood, and that is one of the most nou·ishing foods he can well have. Indeed, for the first day or two after an attack he may be said to be living on his own blood. The patient should be put on a mild unstimulating diet. All that he has should be quite cold. He may be allowed small pieces of ice to suck, but not too much of this, because it is little better than giving him large quantities of water to drink, whereas he should be limited to a pint of liquid in the day at first, gradually increased to two pints. For the first two or three days the diet should consist of iced milk and beef tea jelly, or Valentine's meat essence. And it should be a rule, never to be departed from, that the patient be not allowed to have solid and hot food until the expectoration has been free from blood for one week. It is a useful plan when the patient is convalescing to put large quantities of salt into his beef tea, as he has lost a large quantity of blood, and with it important salts which enter into the composition of the tissues; by so doing this deficiency is supplied. As the patient improves in strength he may get up and take more nourishing food; he may even be given some alcohol if one can be quite sure that the bleeding has ceased for some time. Of course, alcohol at any other time is injurious.

By these means the treatment of hæmoptysis is in the main carried out. No mention has been made of drugs for two reasons; first, they are of doubtful value; and, secondly, for the nurse it is most important to know and carry out the above instructions. It may be mentioned though that one generally in the treatment of hæmoptysis, has in view two ends; first, to allay anxiety and quiet the heart; secondly, to make an attempt to stop the blood spitting. For the first condition morphia and digitalis are generally prescribed; and for the second, some of the so-called hæmostatics are given, such as ergot tannic or gallic acid, acetate of lead, or hamamelis. The bowels should always be kept relaxed, and this is best done by appropriate doses of sulphate of magnesia. At a later period one generally has recourse to hæmatinic, medicine of which iron is the most useful.

*(To be continued.)*

### Croupous Pneumonia.

Paper read before the Nurses' Journal Club at the Training School for Nurses of the Johns Hopkins Hospital, April, 1892.

*(Concluded from page cliii.)*

But how do the leucocytes know that they are needed? On this question, full of mystery, the new learning throws light. It is found that when living bacterial cells are broken down or injuriously acted upon, in the process of dissolution chemical substance is set free from their protoplasm, which as the power of attracting the leucocytes.

This substance is a proteid called myco-protein, or in this instance pneumo-bacterio-protein. It has been isolated and studied. It has definite chemical properties; it is slightly poisonous; it has no curative power; it does not confer immunity; it excites the emigration of the leucocytes and produces fever.

The body tissues also under like conditions evolve similar substances. Experiments are done with turpentine, for instance, by injecting it into the tissues, when a genuine suppuration, the so-called aseptic suppuration, is produced, the white blood corpuscles being drawn to the spot by the substances, called here alkali-albumins, which result from cell degeneration. These wonderful proteids are called positive chemotactic substances.

The word chemataxis is quite new. It is not in the dictionaries, and its meaning must be inferred. From its context in Dr. Welch's lectures it might be translated as the law of attraction between a certain kind of chemical substance and living cells. The subject is absorbingly interesting, for it bears directly on the poisons and counter-poisons of bacterial diseases. Substances which attract cells are positively chemotactic, and those which repel them are negatively chemotactic as regards these cells.

In the pneumonia that we are considering it is the blood serum that degenerates and breaks down the bacterial cells, and sets free the myco-protein, a crushing blow to the bacteria, for their bodies now attenuated by the action of the serum, are diligently eaten up by the leucocytes.

Under the microscope large white blood cells were shown, enclosing in their own substance numerous bacteria, degenerate, dying, and dead.

But the final contest is waged in the blood, and there the true climax is soon to be reached. There, by a series of complex and wonderful chemical changes, appears finally an entirely new substance—one that was not there before—called "anti-pneumotoxine," and this is an antidote to the pneumotoxine. The meeting of the poison with the antidote results in the annihilation of the former, and is signalised by what we know as the crisis.

The crisis, then, is simply the reaction of the system after strenuous effort, and after it, though only for a short time after in human beings, the anti-pneumotoxine may be found in the blood. It is obtained experimentally in laboratory work in a fairly complete degree of purity, and its power as an antidote strikingly demonstrated in work with animals. Experimental work also gives a pretty explanation of the marked chill and high fever that belong to pneumonia.

High temperature is necessary for the successful production of the antidote. Without heat it cannot be formed, and a chill is one of Nature's ways of securing a rise of temperature. Active muscular contractions also produce heat; so that in the chill we see the vasomotor spasm, the contraction of the surface vessels (with consequent massing of blood in the internal organs), and the violent shivering, all acting energetically to get up steam, as it were, to stimulate the nervous system to a tremendous effort. The theory given being that the heat regulating centres are not unbalanced, as has been elsewhere stated in explanation, but rather tuned to a higher key, and thus fitted to control the increased absorption of oxygen and increased combustion.

Then, to review briefly, we see the bacteria enter the lungs and begin forming their poison, the pneumotoxine. The blood serum attacks the bacteria, and has power to harm some, at least, of them enough to set free the myco-protein from their bodies. The myco-protein attracts the leucocytes, they assemble in the lungs, form a consolidation, and eat up as fast it is possible, the bacteria.

Finally, by intricate steps, the antidote against the pneumotoxine appears in the blood and the victory is with it.

Immunity after one attack is not conferred in pneumonia. On the contrary, the lungs, weakened by having been the scene of this fierce struggle for supremacy, are more liable to yield to subsequent invasions.

The antidotal substance is produced only in small amounts and for a short time, but the work of the future will aim at the production and management of the anti-pneumotoxine in such form and quantity as to be an ever ready weapon at hand for our defence.

Nor is this an impossible goal; the way is open, and the outlook is bright.

### Girl Nurses Again.

This subject seems to have roused general interest, for letters dealing with its different aspects continue to find their way to the office. A correspondent signing herself "Mater" writes at some length, but is evidently unacquainted with hospital regulations as to age, or she would hardly assert "It is perfectly true that the great majority of nurses who crowd our hospitals and nursing institutions are immature. They are girls who are playing at nursing." Now although we may meet with such occasionally, they are certainly exceptions. Most readers of The Hospital are aware that the best training schools refuse probationers under twenty-five. Nurses are therefore now usually twenty-seven, and more often twenty-nine or thirty years of age before they rank as fully trained nurses. "Mater" quotes the unfortunate experience of a friend who "telegraphed to town, a short time since, for two nurses for a case of illness in her family. Two young ones arrived, and my friend described them as simply 'atrocious.' In another case, the nurse sent for did not even know how to measure out the medicine, and was so ignorant, yet self-complacent withal, that she was sent off the next day. Indeed, cases of glaring inefficiency of the young nurses who are forcing themselves upon us and pushing out experienced private nurses by force or numbers are daily apparent." The writer forgets that the remedy for this lies in her own hands. When applying for nurses she should stipulate for fully trained ones of a stated age. To call anyone a nurse who does not know how to measure medicine, is obviously absurd, and an employer should complain to the institution which supplied her. Again, nurses "forcing themselves upon us" is hardly a possible contingency. No one is obliged to take any attendant. Surely persons who prefer elderly private nurses can continue to employ them, leaving the younger "certificated" women for those whose choice lies in that direction. The really good experienced nurse is not likely to be "pushed out" of the position which she has gained, by any influx of younger women, and we venture to think that there is still room for both classes of nurses in a profession which is daily increasing in importance. When infirmaries and other institutions follow the example of the metropolitan hospitals we shall hear no more of "girl nurses" but only of young women in the best working years of their lives.

### International Nursing Congress.

MRS. BEDFORD FENWICK'S POSITION.

WITH reference to the information regarding this Congress, which appeared in these columns on December 3rd, 1892, January 28th, and February 11th, 1893, we understand that Mrs. Bedford Fenwick when in America suggested a Nursing Congress, and that it is probable that she intimated her willingness to act as chairwoman of the British Section when she talked the matter over with Mrs. Flower. When, however, the question came to be formally discussed by those responsible for the arrangements, it was determined to organize the Congress as originally formulated by the International Congress of Charities, Correction, and Philanthropy at their meeting n July, 1892. It appears that probably Mrs. Flower did not notify Mrs. Fenwick of this, and so Mrs Bedford Fenwick might have some grounds for saying that she has been badly treated, but in any case she has assumed more than she had any right to do. The following correspondence may prove interesting to many readers.

THE POSITION IN ENGLAND.

On January 26th we wrote to the Chairman of the Congress at Washington, as follows:—

The Editor of THE HOSPITAL presents his compliments to the Chairman of the International Nursing Congress, Chicago, and begs to enclose:—

1. A letter of the 13th inst. addressed by Mrs. Bedford Fenwick to some matrons and others in charge of hospitals and nursing institutions in this country.
2. Extract from a paper read before the Royal British Nurses' Association on the 20th inst. by Mrs. Bedford Fenwick.
3. Copy of a letter from Miss Lankester, dated 17th inst.

The Chairman will perceive that in Mrs. Bedford Fenwick's letter of the 13th inst. it is stated that she "has been requested by Mrs. J. M. Flower, the President of the Illinois Training School for Nurses and Chairwoman of the Women's Branch of the Social and Moral Reform Congress, to act as Chairwoman of the British Section of the Nursing Congress, which will be held at Chicago in June next;" that Miss Lankester states that "Mrs. Bedford Fenwick, the representative of Nursing on the Ladies' Committee of the British Royal Commission, has been requested by Miss Hampton, the Chairwoman of the Nursing Section of the United States, to organise the British portion of the forthcoming Nursing Congress in Chicago;" and that Mrs. Bedford Fenwick, in her paper, states: "I have had the honour of being requested by Mrs. Flower and Miss Hampton to organise the British Section, and with the help of the Sub-Committee on Nursing, in connection with the Royal Commission, I have already received offers of more papers than, I fear, there will be time to read and discuss."

The Editor telegraphed to the Chairman as to the statements made by Miss Lankester in regard to Mrs. Bedford Fenwick, the correctness of which the Chairman has contradicted by cable. The Chairman will perceive that the divers and contradictory statements which have been made are calculated to create difficulties and may, unless they are officially confirmed or refuted, militate against the success of the Conference by creating endless confusion. Will the Chairman, therefore, kindly cable replies to the following questions:—

1. Who originated the International Nursing Congress at Chicago?
2. Has Mrs. Bedford Fenwick (or anyone else) been requested officially or otherwise to act as Chairwoman of the British section of the Nursing Congress which will be held at Chicago in June next?
3. Has Mrs. J. M. Flower the power to nominate anyone as Chairwoman of the British section of the Nursing Congress, and has she requested anybody to act in that capacity in this country?

The Editor of THE HOSPITAL would be further obliged if the Chairman could send, in addition to the cable, an official answer in writing to this letter, containing all necessary explanations, so that it may be published in THE HOSPITAL without delay. Anything that the Chairman can do to remove the present state of misapprehension and uncertainty will certainly be much appreciated by the responsible heads of the principal nurse training schools and hospitals in Great Britain and Ireland.

THE POSITION IN AMERICA.

The Chairman of the Section on Hospitals, &c., wrote in reply as follows, from Washington, D. C., February 8th, 1893:—

Sir,—Your communication of January 26th, with enclosures, is received. I cannot speak positively as to what may have occurred prior to my acceptance of the position as Chairman of the Section of the International Congress of Charities which relates to the hospital care of the sick, training schools, dispensaries, and first aid. I believe, however, that Mrs. Fenwick, when in Chicago in October last, suggested the formation of an International Nursing Congress and at that time was asked by Mrs. Flower to be Chairman of the British Section, but later on, after discussion, it was decided by the General Committee that a separate Nursing Congress was not expedient, but that it should form a sub-division under the general control of the Section on Hospitals, &c.

When I accepted the position as Chairman of the Section, it was with the distinct understanding that Dr. Hurd, of the Johns Hopkins Hospital, should be appointed secretary, and Miss Hampton, chairman of the sub-section relating to Nurses, and that we should proceed to organise the work, prepare a programme, issue invitations, &c., untrammelled by anything that might have been said or done prior to our appointment (which conditions were accepted by the General Committee), and this we have proceeded to do. Neither Miss Hampton nor myself have requested Mrs. Bedford Fenwick (nor anyone else) officially or otherwise, to act as Chairwoman of the British Section of the Nursing Conference; we know nothing of any such office. I am confident, also, that since the organisation of the Section, Mrs. J. M. Flower has made no such request or nomination.

Invitations have been sent to each training school of whose existence we know—to send delegates, to present papers, and take part in the discussions. All are to be treated exactly alike, and as independent organizations. We are aware that there are differences of opinion on various points among the authorities of English training schools, among English nurses, and among those interested in such schools and nurses; and that to recognize any one person as the representative of British nurses or of British training schools would defeat one of the main objects of the gathering.

We do not undertake to decide between the parties in controversy. We invite all to come and take part in the discussion of matters relating to hospital and nursing interests, and to put personal controversy entirely aside for this occasion. It is to be hoped that such a meeting on neutral ground and a perfectly equal footing, may prove be the first step towards a better understanding, and to reconciliation of conflicting views and interests.

### Everybody's Opinion.

### A REASONABLE REQUEST.

"SISTER-IN-CHARGE" writes: Seeing appeals in your paper for various things I venture to ask if you will be good enough to insert a request for a small harmonium or American organ for the little hospital at Blaenavon, Monmouth. There are eight beds for men, only accidents being taken in. It is a wild mountainous district, and we get no kind visitors as other places of the kind do. It is, therefore, very dull for the patients, who are quite uneducated, but very fond of music, for which they often ask. I have read and valued THE HOSPITAL during the six years that I have been a nurse.

### PROVINCIAL TRAINING.

A LIVERPOOL NURSE writes: It is wearisome, to say the least of it, to hear some London trained nurses, working in a provincial hospital, continually boasting of their training in the great City. I will certainly admit that many of the best hospitals are in London, but st ll there are hospitals and training schools in the provinces that could easily compete with some of them. A nurse, who has gone through a course of training in a small London hospital, takes a post as nurse or sister in a provincial institution, calls herself a "London Trained Nurse," and, to quote Algernon Sidney's words, "Apes a superiority which exists merely in her own conceit." Why, because they are London trained, do they come and abuse our provincial schools?

### YOUNG NURSES.

"Nurse C." writes: There seems to be a great discussion going on about girl nurses. I myself began to train before I was twenty, but in a hospital for children only. I have since trained for adult work, I think it is the greatest mistake for any one to go into a hospital under twenty-one. At that age a girl might enter a hospital for children, and a great deal depends, of course, on the individual and on the way in which she has been brought up. If a woman has to earn her own living, it is rather long to wait until she is twenty-five, and she does not often care to try two professions. Many girls might safely enter at twenty-three, but a matron should be allowed to use her own judgment as to the kind of person she takes. Some people think children's work a waste of time, but I consider it advantageous. I have had many probationers over twenty-five in adult wards that I should be sorry to leave in charge of any patient, and have found many younger ones able to take full control. So many enter hospitals without fully understanding what they undertake, apart from possible injury to their constitution. I love my work, and would not change for any other, but I would never allow anyone, if I could, to go into a hospital at the age I entered, and I hope more persons will keep to the rules adopted by the leading hospitals as to age.

### AMERICAN EXPERIENCES.

"A. E. I." writes: In answer to "An Interested Nurse," I send a few details which may prove useful to her and to other readers of The Hospital In the first place, with regard to what to take. Boots and underlinen are cheaper and better in America than here; but dressmaking, &c., is very expensive over there, and gloves are a ruinous price. If a nurse has the moral courage to stick to her uniform (in a country where uniforms of all kinds are looked on with disfavour, and almost as a badge of disgrace, or at least of inferiority), she will be wise, for it will save her endless expenses. There are no lodgings to be had in New York, and I believe not in America. You must either board with a family or take a room "for light housekeeping," which means that the room will be furnished as a sitting-room, with a folding bed to be put up in the day time. Beds assume a variety of shapes in America during the day—pianos, writing desks, bookshelves, and many others, both simple and complex. Rents are dear, a small bed-room costing $3 or twelve shillings a week, but food is cheap. When you have a room for "light housekeeping" it generally contains a large cupboard for dry stores. You procure an oil stove and make your morning coffee, which with new rolls and an egg generally constitutes your breakfast. Mid-day dinner you can get at a restaurant; there are a great variety of restaurants and you can have a good dinner from 25 cents upwards. If you order a steak you find bread and potatoes are included. Your evening meal you can arrange like your breakfast, and no one thinks of having more than three meals a-day. You can board fairly well for six or seven dollars a-week (24 to 28 shillings.) I speak for those who simply want respectable rooms and food, prices at fashionable boarding houses being enormously high. The meals in boarding houses are abundant but slightly monotonous. Steak is the American's great stand-by; I have had steak for breakfast, dinner, and supper, and you are sure to have it in a boarding house fourteen times a-week. Breakfast consists of oatmeal, steak, fried or stewed potatos, and fruit. Dinner: steak, potatos, tomatoes, and perhaps two or three other vegetables; and dessert in America nearly always means apple or pumpkin pie made like enlarged mince pies, or some kind of pudding. Supper: chops, stewed fruit and cakes. The variety from steak is hash, consisting of salt beef and potatoe mixed together and is rather nice. In winter griddle cakes are served with syrup for breakfast instead of oatmeal. All bread is served new, and often quite hot. Tea appears at each meal, and seems very nasty until you get accustomed to it; if you want English tea you have to ask specially for "English breakfast tea." Americans eat butter with everything. Ice cream is often given instead of pudding, and iced water is poured out at every meal, and sometimes a glass of milk besides. There are houses in New York where only nurses are taken; they have to attend to their own rooms, except for a weekly cleaning. Travelling in America is easy, you can check your luggage through from the house you are at to the house you are going to. You receive a metal check, and the boxes are not given up until you produce it. A hand-bag that you can carry yourself is a necessity, as porters are scarce, and cabs charge exorbitantly. The "surface cars" (trams) run constantly from all points at the uniform charge of 5 cents. (2½d.), you can go a "block" (from one turning to the next) or you can go as far as the tram runs for 5 cents. The coinage is easy to learn, but deceiving, because 2 dols., for instance, looks so much less than 8s. Private nursing is well paid; $20 (£4) a-week, and Americans are simply charming in their own homes. I was private nursing for over three years, and was always treated with the utmost consideration, more as a guest than a paid worker. I made many friends, and shall always feel that I owe to Americans a great debt of gratitude for the kindness shown to a stranger. The doctors treat their nurses courteously, and are thoughtful for their comfort. An American doctor once said to me, "A good nurse cannot be paid for, but a bad nurse is dear at any price." I should advise a nurse going to take up private work in America to secure one or two introductions to doctors. American nurses naturally do not welcome strangers in their midst, and it is difficult for a foreigner to make her way unaided. Sometimes two or three nurses club together and take a small unfurnished flat, taking it in turns to stay at home and do the housekeeping. Washing is frightfully dear.

### Royal British Nurses' Association.

A lecture on Cholera Nursing was given on the 17th inst. by Miss Annesley Kenealy, a member of this association. The chair was taken by Dr. Bedford Fenwick, and Dr. Gage Brown was also on the platform. Miss Kenealy paid a short visit to Hamburg during the epidemic of last year, and she described the Eppendorf Hospital, to which she was temporarily attached. There was no matron, the nursing being superintended by an elderly male inspector, and there were male attendants for the men. When patients suffered from very severe bed sores they were slung in canvas hammocks in a perpetual bath of warm running water. Their diet was rather peculiar, consisting of black coffee, raw beef, veal, &c., but the patients seemed to do well on it. There was but little discipline in the wards, and the nurses wore no caps, and were not particular about uniform.

### Appointments.

Miss M. E. Jones has been appointed Matron of the Eastern Hospital (Metropolitan Asylums Board). Miss Jones was trained at the Royal Southern Hospital, Liverpool, was Matron of the Fever Hospital, Middlesborough, and has held other appointments.

Miss Katherine Elphick has been appointed Lady Superintendent and Matron of the North London Consumption Hospital. Miss Elphick was trained at King's College Hospital, was afterwards sister at the Children's Hospital, Pendlebury, and for the last eighteen months has held the post of sister at the Children's Hospital, Great Ormond Street. We wish Miss Elphick every success in her new work.

Liverpool Royal Infirmary.—We are glad to be able to announce that Miss Charlotte M. Bann was appointed Lady Superintendent of this hospital on the 18th inst. No better appointment could have been made. Miss Bann has done most excellent work at the Bristol General Hospital, having previously been Matron of the Swansea General Hospital, and subsequently of the General Hospital, Birmingham. Miss Bann trained at the Manchester Royal Infirmary, and her record is uniformly good. We congratulate the Committee upon the appointment they have made, as it is well calculated to promote and extend the usefulness and efficiency of the Liverpool Royal Infirmary.

### An American Training School.

THE *Public Ledger* of Philadelphia reports on the "Sixth Annual Graduating Exercises" of the Nurses' Training School of University Hospital, which took place last month. Diplomas were granted to fourteen young women, and Dr. Billings remarked that the average figures of their examination papers were decidedly better than last year. Of the thirty-one candidates who had been received on a month's probation during the last twelvemonths eighteen were accepted for training, and three junior and five senior nurses were discharged for various reasons. Requests for rules, &c., having come from 500 persons, 111 were finally chosen from which to select candidates for the present year. Twenty-four applications had been received from other hospitals for graduates to fill various posts, some of these being at a considerable distance from Philadelphia. The average number of nurses on duty during the year at the University Hospital is given as thirty-five, including probationers."

Mr. Wood congratulated those graduates who received diplomas on having been trained at an institution which is considered one of the best in the country. Dr. John Ashhurst made a good speech, comparing the romantic with the practical side of nursing. He gave the nurses some very sound advice when he bid them, on going into other hospitals, "to be slow to antagonise traditions they found there"; and, again, "the duty of a nurse always is to be loyal to the physician to the best of her ability." He also urged his hearers neither to think too little of their efforts, nor to claim too large a share of credit, and, in conclusion, "not to be specialists, but to be able to nurse any case to which they were called."

### For Reading to the Sick.

### EXAMPLE.

IT is very cheering and helpful when we are despondent and downhearted to think of one who has passed through similar affliction with ourselves, and to note how hopefully he has borne up against his imperfections and anxieties, and done his work nobly in spite of bodily sufferings and trials. We long to be like him, but think it hopeless that we should ever be cheerful under his circumstances, much less under our own, until we begin to think of what can be the cause of such brightness and contentment.

First of all, there is the example of our Lord Himself, who was a man of like passions as ourselves, only without sin, who bare in His own body all the miseries and sins of all mankind, and yet said, "Father, Thy will be done." Then His great apostle, Paul, comes to our minds, whose endurance, efforts, labour, service, and self-sacrifice for God's cause has made his own name shine among the faithful followers of Christ. How grievous is the tale of his sufferings during his long life! How we should pity ourselves were we in a like plight! He tells us he had been beaten five times by the Jews and three times by rods, and been shipwrecked three times; had been in perils of all kinds, both among the heathen and his own countrymen, while weariness, pains, watchings, hunger, thirst, fastings, cold, and nakedness fill up a list not easily matched. Yet, for all this, he rejoiced and gloried in the infirmities which rendered him more meet for God's kingdom. It was his wonderful faith and love which supported him, and his unselfishness, his God-given resolution to spend and be spent for the good of others that gave the key to his conduct. It is for ourselves to say how nearly we may copy St. Paul. If we bear our sufferings in the same spirit and with the patience shown by him we may attain to a like perfection with him. His own shortcomings before his conversion taught him many a lesson after. The Apostle had been a persecutor, so his own persecutions brought home to him the harshness of his former conduct, and rendered him humble and gentle.

Many a Christian has been heartened up to join in the train of those who "follow the Lamb whither soever He goeth"—not always to the stake or sword, to lay down their lives for the faith, but to bear without murmuring a lot which cuts them off from their usual pleasures and employments. Alone with their God they fight out the battle against self and Satan, and as He who is with them is mightier than their enemies they conquer in the strife.

### Notes and Queries.

### Queries.

(42) *Patent Office.*—Can any of your readers give me address of the Patent Office?—*Videns.*

(43) *Convalescent Home.*—Can any reader tell me of a place (seaside or country) where a home for invalids, established by trained and experienced nurses, would have fair prospect of success?—*Nurse J.*

(44) *Physiology.*—I should be glad to hear of one or two nurses who would join a class in this subject held by a competent lady teacher.—*Nurse E. F.*

(45) *Bournemouth.*—Can any one tell me of a nursing institute for private nurses at Bournemouth?—*A Private Nurse.*

(46) *College Cap.*—Can any person wear a college cap without infringing the law? and can nothing be done to prevent those who are not trained nurses from wearing uniform?—*Mirfield.*

(47) *Temperature Charts.*—Can anyone tell me where to get temperature charts made up in books of 50 or 100?—*M. R. P.*

(48) *Breakages.*—Is it usual for nurses to pay for breakages in a nursing home when no agreement was made to do so?—*N. W.*

(49) *L.O.S.*—I should be glad to know if I could read up by myself for the L.O.S.—*Nurse Edith.*

(50) *Diphtheria.*—In how many London hospitals, general and children's, is there an isolated ward for this disease?—*E.*

(51) *Cottage Nursing.*—A useful book on this subject required by *Nurse Eva.*

(52) *Second-hand.*—Can you tell me where to obtain "Tanner's Index of Diseases" second-hand?—*Nurse A.*

(53) *District Nursing.*—Could you kindly inform me where a suitable book for reporting the work of a district nurse can be bought?—*S. R.*

(54) *Lancaster.*—Can you tell me of a useful book on district nursing?—*W.*

(55) *Deaconesses.*—Can you tell me where I can learn particulars of Deaconess' Homes in England?—*A. M.*

### Answers.

(42) *Patent Office* (*Videns*).—Patent Office, 25, Southampton Buildings, W.C.; office for sale of forms, 38, Cursitor Street, E.C.; British and Colonial Patent Office, 151, Strand.

(43) *Convalescent Home* (*Nurse J.*).—We shall be glad of suggestions on this subject from our readers. Some homes do not admit invalids who require trained nurses, and probably an opening could be easily found for a convalescent establishment conducted by experienced nurses.

(44) *Physiology* (*Nurse E. F.*).—Probably several ladies, not necessarily nurses, would be glad to arrange for this.

(45) *Bournemouth* (*Private Nurse*).—Victoria Home, Bournemouth.

(46) *College Cap* (*Mirfield*).—We are not aware of any law existing in the first case. In the second, we wish that good taste prevented persons with no claim to the honourable title of nurse from affecting the costume. England is a free country!

(47) *Temperature Charts* (*M.R.P.*).—Convenient books containing 50 charts are published by Daniel, Son and Co., 52, Beaumont Street, W. Each page takes three weeks. Price 1s. per book. Messrs. Allen and Hanbury, Plough Court, Lombard-street, supply a book for 6d. containing 30 charts, four weeks each. Wodderspoon and Co., 7, Serle Street, Lincolns Inn, also supply charts.

(48) *Breakages* (*N.W.*).—We imagine paying for breakages should be a matter of mutual agreement when the nurse is engaged. It could hardly be insisted on otherwise.

(49) *L.O.S.* (*Nurse Edith*).—We think you could study with this object, but you would have also to secure the practical experience. The books you ask about are "Manual Midwifery," by Cullingworth, "Obstetric Hints for Midwives," and various other works.

(50) *Diphtheria* (*E.*).—To obtain accurate information on this point, you had better find all the metropolitan hospitals in "Burdett's Hospital Journal," unless you are already acquainted with them, and apply to each for the information you require.

(52) *Second-hand* (*Nurse A.*).—At almost any second-hand medical bookshop, or at Kimpton's, in Holborn.

(53) *District Nursing* (*S. R.*).—You can get books at Lowe's, 157, High Holborn. He supplies the Q.V.J.I.

(54) *Lancaster* (*W.*).—Mrs. Dacre Craven's book on District Nursing is probably one you would find suitable.

(55) *Deaconesses* (*A. M.*).—You will find addresses of nearly a dozen in "The Women's Year Book," which you can buy for 9d.

### Hints to Nurses.

DR. R. PROSSER WHITE'S "Combined Nursing Report and Diet Table" is likely to be useful to private and district nurses. It is marked out in a clear, simple fashion, easy to fill in, and satisfactory in every way. These "report papers" can be procured from Benson and Co., 10, Darlington Street, Wigan. No price is given.

### Minor Appointments.

MISS FRANCES PARKES has been appointed Head Nurse at the City Asylum, Birmingham, having been Deputy Head Nurse for eighteen months, and previous to that Charge Nurse for seven years. Miss Parkes was trained at Oxford Asylum

### The Muses' Looking Glass.

### BURNE JONES AT THE NEW GALLERY.

Readers of Mr. Du Maurier's dream novel of last season will remember with what joy the convict hero lay down after his monotonous days of work and prepared to enter the dream world to which he had obtained the clue. Something of the same feeling assails the visitor to the New Gallery at finding himself in these rooms, dedicated to Burne Jones his art. It is a dream world into which he has entered, but the dreams are no common medley of thin shadows; they are the visions of a poet and a seer. Nothing is quite real in this world, not even the portraits with names attached. We are sure that Miss Margaret Burne Jones never sat "cutting bread and butter" in just that set of bones and flesh. Little Philip Comyns Carr never could have messed that grey pinafore and been scolded for leaving his toys about with that very wistful face, which seems to float on the surface of the canvas. We are quite confident that the artist had all to do with draping those green ladies who sit by green water on green grass in a green summer. This is a land of illusion and glamour, and if the nineteenth century intrudes itself at all, it modestly takes a back seat and surrenders the honours to its far ancestors.

And yet, whence do the women get that expression in their eyes? In every direction, from every canvas, these faces compel attention. "They are bored and weary of their lives," says the critic of the *Daily News*, "we do not want our women to look like that." "And thank goodness they don't," says critic No. 2; while critic No. 3 stands arguing that they have no expression at all. Critic No. 4, in the *Speaker*, is mainly irate because he cannot tell the water-colours from oils; and critic No. 5 stands declaiming to a group of disciples in the large room, "If you want back-grounds, here you are, don't you know." Leaving the backgrounds and the water-colour dispute alone, let us go back to the women's faces. It is given to very few men successfully to ante-date themselves. Whether a man will or no, the seal of his own times is generally to be found impressed on his work. Mr. Burne Jones has painfully cut himself aloof from modern convention of conception, treatment, colouring, and technique in his art. It is hardly too much to say that this collection gives no hint in any one accessory of the century in which it was produced. And behold, the nineteenth century, baffled at all points, has taken a choice revenge. In the very secret place of the artist's soul, in the eyes of the race of women his genius has created, the spirit of the age is triumphant. Not the restless critical male spirit; that has no place here either in woman or man. The men, indeed, are mainly wrapt in contemplation of woman. With one or two exceptions they are lost in dreams of love and look no further. But the women are all looking out of themselves and beyond, and the glory of the invisible reflected upon them, outshines the glamour of the actual. They have tasted of the tree of knowledge, and their eyes are opened to behold the nakedness of their fellows. These women have lived no happily shielded lives in ignorance of ill. A shadow of infinite sorrow, not their own, rests on their brows; there is pity and high resolve and the faith which yearns and conquers in their eyes. The lips have lost the tender lines of resignation which sadness brought to the women of old whose lot was to sit still. The tenderness lingers still round the firm full curves of mouth and chin, but it is the tenderness of power and purpose. They are of one family, these women, gracious race with many points in common, and this double perception more than all of the ideal and the real. And yet studied carefully each one bears the stamp of a personality all her own.

Here is Beatrice, for instance, in soft red garment and white hood falling a little back from her hair, passing soberly down the street, unconscious of the admiring women in the doorway. The pure, steadfast outline of the face seen in profile, reveals a nature wrapt in heavenly contemplation. But here is no saint's nature, shrinking from earthly contamination. This is the face of the brave woman who left the choirs of Paradise and penetrated into hell itself, that she might save the soul of her lover.

The symbolic figure of Caritas folding her blue robe round the two babies nestling in her arms, is full of a very beautiful pity; four other little ones are clustered round her feet, one playing hardily with her red sash, while another is eating one of her golden fruits. Charity may well be joyful as she feels the children's hands about her. Yet, if her face is glowing, the eyes are filled with unshed tears. Like her we look beyond the beautiful framework, and see the multitude in our very midst of little sufferers, whom Charity's arm is too short to reach, her robe too scanty to embrace.

Not all the women have the touch of care to which the critics object. The very perfection of ripe and beautiful womanhood is seen in the Flamma Vestalis and Sibylla Delphica. In some faces, too, on the Golden Stairs and in The Mill, the far-seeing look is lost for the moment in the gladness of the present. It is in the love scenes that this abstractedness is best studied. And here in one or two instances it is conspicuous enough to justify the young man's complaint in the *Strand Magazine* that "a woman who takes an interest in things outside herself becomes a nuisance." We are induced to think, though, that these worshipping knights were not conscious of this far-away gleam in the eyes of their loves. We fancy that the lover who rests his head so contentedly on the soft hair of that girl in "Love among the Ruins," does not guess that while her arms are clinging about him, her face is half turned away in some dreamy realization of the mystery of desolation which surrounds them both.

In the "Chant d'Amour" both figures are the prey of the slumbering Cupid concealed behind the instrument of music. But to the woman love is winged, and carries her out into far realms of fancy; for the man it means just that one woman singing her soul out before him.

So in the "Lans Veneris" all the five knights looking in at the window are distracted with love in their different ways. The women are evidently "taking an interest in things outside themselves"—not, we regret to notice, exclusively knights.

The most remarkable example of all is King Cophetua and the Beggar Maid. Here we have the King sitting nursing his crown in an attitude of wrapt adoration at the pearl he has rescued from the dung-hill. The contemplation of her rags adds to his contented delight in the beauty which shines through them and in his own discernment. He has not a thought beyond himself and her. But she sits above him in her poor attire, a queen in truth, but not of his making; accepting his homage with unconscious grace, and gazing into the future with eyes which never even glance at the riches he is ready to pour out at her feet. So might Esther have sat when she linked her life to that of the heathen king with purpose to save her race.

We have dwelt on this aspect of the artist's work, perhaps, too exclusively. We conclude with some mention of the picture where women figures claim no attention. It is the figure "of a knight who forgave his enemy when he might have destroyed him, and how the image of Christ kissed him, in token that his acts had pleased God." He is kneeling bareheaded in a little woodland shrine to which access is gained by a wooden stair and a latchet gate. Through the open sides the figure of his squire is seen waiting with his master's horse in the field below. Beyond is a glimpse of a dark wood and the narrow path through which he has been guided to the shrine. The sun is streaming from the left of the picture, lighting up a distant glimpse of the sky and touching the knight's armour with bright points of light. It rests, too, on the figure of the Christ, which stretches forward from the crucifix on the wall to embrace the knight. It is a wooden figure, galvanized into a life miraculous, rather than strained or unnatural, and the hands rest lovingly, as human hands might rest, on the knight's shoulders, and the knight kneels motionless, resting in the assured presence of his very Master, as one who has passed the bitterness of death. For it is no light injury which has graven itself on his face; the enemy has done his work well; he has emptied his life of all but the fierce thirst for vengeance, and the battle which ended in forgiveness has left the heart still sore and empty. But now he sees of the travails of his soul, and is satisfied. A glowing foreground of scarlet flowers surrounds the shrine and completes the symbolism.

